# Early changes in microRNA expression in Arabidopsis plants infected with the fungal pathogen *Fusarium graminearum*

**DOI:** 10.1371/journal.pone.0318532

**Published:** 2025-02-06

**Authors:** Savio S. Ferreira, Suman Pandey, Jesseca Hemminger, Serdar Bozdag, Mauricio S. Antunes

**Affiliations:** 1 Department of Biological Sciences, University of North Texas, Denton, Texas, United States of America; 2 BioDiscovery Institute, University of North Texas, Denton, Texas, United States of America; 3 Department of Computer Science & Engineering, University of North Texas, Denton, Texas, United States of America; 4 Center for Computational Life Sciences, University of North Texas, Denton, Texas, United States of America; 5 Department of Mathematics, University of North Texas, Denton, Texas, United States of America; University of Agriculture Faisalabad, PAKISTAN

## Abstract

Plants respond to biotic stressors by modulating various processes in an attempt to limit the attack by a pathogen or herbivore. Triggering these different defense processes requires orchestration of a network of proteins and RNA molecules that includes microRNAs (miRNAs). These short RNA molecules (20–22 nucleotides) have been shown to be important players in the early responses of plants to stresses because they can rapidly regulate the expression levels of a network of downstream genes. The ascomycete *Fusarium graminearum* is an important fungal pathogen that causes significant losses in cereal crops worldwide. Using the well-characterized *Fusarium-Arabidopsis* pathosystem, we investigated how plants change expression of their miRNAs globally during the early stages of infection by *F*. *graminearum*. We have created a catalog of miRNAs that have differential expression in infected samples even before any visual symptoms of the infection are present. In addition to miRNAs that have been previously implicated in stress responses, we have also identified evolutionarily young miRNAs whose levels change significantly in response to fungal infection. Some of these young miRNAs have homologs present in cereals, which suggest that some of these miRNAs could be drivers of stress response. By examining if the miRNAs in this catalog have causal roles in plant infection response, a unique path toward development of plants with increased resistance to fungal pathogens can be developed.

## Introduction

Plants have developed exquisite ways to constantly survey their environment and respond accordingly to different stress conditions. These responses typically involve many layers, from activation of membrane-bound receptor proteins to up- or down-regulation of expression of genes that ultimately lead to a physiological change to cope with the stress. One such layer consists of different types of non-coding RNA molecules, which can control expression of a downstream network of genes involved in the response to a particular stress.

Small RNAs (sRNAs), such as microRNAs (miRNAs) and small interfering RNAs (siRNAs), are 20–30 nucleotide-long non-coding RNA molecules that are involved in the sequence-specific regulation of gene expression at the transcriptional or post-transcriptional level. In addition to their roles in growth, development and maintenance of genome integrity, sRNAs are also important components in plant stress responses. Changes in the levels of endogenous plant sRNAs in response to external stimuli, such as pathogen infection, environmental conditions, and nutrient availability, are well-documented [1–3]. These sRNA regulatory networks in plants are thought to have evolved as a cellular defense mechanism against RNA viruses and transposable elements that were later adapted to regulate the expression of endogenous genes [4]. Abundant evidence now suggests that endogenous plant sRNAs and their activity in silencing target genes may serve as a general regulatory mechanism in plant immune responses to many pathogens [5–7]. In fact, to try to counteract these responses, some bacterial effector proteins and viral proteins have been shown to suppress host miRNA biogenesis and/or activity [8–10]. Therefore, several studies have looked at changes in the levels of plant sRNAs, and how they affect the protein-coding transcriptome, to identify common mechanisms involved in responses of plants to pathogens and pests [11, 12].

In plants, several miRNAs have been shown to be up-regulated, whereas others are down-regulated, during their interaction with pathogens and pests [13–16]. Many of these miRNAs are predicted to function as early regulators of stress-induced transcription factors that, in turn, influence the expression of defense genes [17]. For example, the levels of specific miRNAs have been shown to change during progression of the Huanglongbing (HLB) disease in citrus [18], infection of Arabidopsis plants by the bacterial pathogen *Pseudomonas syringae* [19], and the infection of Lilium plants by the fungal pathogen *Botrytis elliptica* [13]. Furthermore, differential regulation of miRNAs in plants has been observed depending on whether they interact with a pathogen or beneficial organism, with differences also observed between roots and leaves [14].

Previous recent studies on the interactions between biotic factors and Arabidopsis plants have mostly focused on changes in protein-coding genes by RNA-Seq analysis [20–24]. Changes in sRNAs are usually not considered, although a bioinformatics approach has been applied to identify miRNA promoters in Arabidopsis that are putatively bound and regulated by *Xanthomonas campestris* effector proteins [25]. Although this study identified miRNAs that may be up-regulated by bacterial effector proteins, it did not provide information on potentially down-regulated miRNAs or which of these miRNAs would be up-regulated in the initial stages of infection. In another study, miR393 was shown to be repressed in response to lipopolysaccharide (LPS) treatment [26]. LPS is a major component of the outer membrane of Gram-negative bacteria and is a potential inducer of plant defense responses. In this case, a single miRNA was identified in the response, making it unclear whether this response is specific to Gram-negative bacteria infection or a more general stress response.

*Fusarium graminearum* is an ascomycete fungal pathogen, the causative agent of Fusarium head blight (FHB, also known as scab or ear blight) disease in wheat and barley, and ear and stalk rot disease in maize. FHB causes significant yield losses, which can exceed 50% when conditions favor the disease; however, it poses a more significant threat to grain quality and animal and human health [27]. As the disease progresses, *F*. *graminearum* produces mycotoxins, such as the trichothecene toxin deoxynivalenol (DON) and the oestrogenic mycotoxin zearalenone (ZEA), which result in reduced grain quality [28]. Consumption of food and feed produced from toxin-contaminated plants can have serious adverse effects on human and animal health [29]. Currently, there are no wheat cultivars with resistance to FHB, and limited information is available on the mechanisms employed by the host plant to respond to the disease, as well as how the fungus targets host physiology to promote infection. Previous studies of genome-wide expression changes in plants infected with *F*. *graminearum* have focused on changes in protein-coding genes, while small non-coding RNAs were not assessed. These studies identified changes in defense related genes, as well as genes involved in primary metabolism, photosynthesis and transcriptional regulation [30]. Therefore, there is a need to better understand how plants respond to infection by this pathogen.

The *Fusarium*-*Arabidopsis* interaction has become an important model pathosystem for characterizing the molecular and physiological basis of plant response to *F*. *graminearum* [28–30]. The number of currently annotated miRNA genes in *Arabidopsis thaliana* (approx. 430) is significantly higher than in the cereal species this fungus normally infects [31]. Here, we investigated changes in plant miRNA expression levels during the early stages (*i*.*e*., prior to the onset of visible disease symptoms) of *F*. *graminearum* infection of *Arabidopsis thaliana* leaves to understand the early plant responses elicited by this fungus. We have produced a list of miRNAs that have significant differential expression in early stages of *F*. *graminearum* infection compared to the mock-inoculated (*i*.*e*., control) samples. We observed that the known targets of these differentially expressed miRNAs are involved in several biological processes related to stress response. Particularly, we identified two miRNAs, namely miR855 and miR826a, which are likely early regulators of stress response and warrant future research to examine their roles and potential use to develop disease resistant crops.

## Materials and methods

### Plant growth

*Arabidopsis thaliana* Col-0 seeds underwent surface sterilization using 10% commercial bleach before being planted on Murashige and Skoog (MS) agar plates. Subsequently, the plates were stored at 4°C for two days and transferred to a growth chamber (Conviron ATC26) with short-day (10-h light/14-h dark) cycles at 150 *μ*mol m^−2^ s^−1^ light intensity, 22°C and 60–70% relative humidity. After 14 days, seedlings were moved to pots filled with Sunshine Mix #1 (Sun Gro Horticulture) and cultivated under identical conditions for an additional 3 weeks.

### Cultivation of *Fusarium graminearum* and plant inoculation

Pathogen cultivation and inoculation was carried out as described in Nalam et al. [32]. Briefly, *Fusarium graminearum* isolate Z-3639 was grown in ½ strength Potato Dextrose Agar (PDA) plates for 8–10 days at 28°C. Plates were flooded with 10 ml of sterile Milli-Q H_2_O and mycelia was scraped with a plastic cell spreader, carefully avoiding scraping the media. The mycelia suspension was then filtered in four layers of sterile cheesecloth, which was washed with an additional 5 ml of sterile Milli-Q H_2_O. Arabidopsis plants, approximately 5 weeks old, were inoculated with the aid of a 1-ml needleless syringe, by infiltrating the whole leaf surface on the abaxial side, totaling 4 leaves per plant. In parallel, additional plants were mock-infiltrated with Milli-Q H_2_O only. Five plants were inoculated per treatment.

### RNA extraction, library preparation and sequencing

Two infiltrated leaves from each plant (mock or fungus inoculated) were harvested into a 1.5 ml microcentrifuge tube as a single biological replicate, totaling five replicates per treatment. Four treatments were used: 3-dpi, mock inoculated; 4-dpi, mock inoculated; 3-dpi, fungus inoculated; 4-dpi, fungus inoculated. Samples were flash frozen in liquid N_2_, followed by grinding with a sterile pestle. Total RNA was extracted from all 20 samples with TRIzol (ThermoFischer Scientific), following standard protocol. For small RNA library preparation, RNA was quantified with QuBit (ThermoFisher Scientific) Broad Range kit and RNA quality was checked the High Sensitivity RNA ScreenTape Analysis (Agilent). Libraries were generated with the QIAseq miRNA Library Kit (Qiagen) and sequenced using a NextSeq 550 High Output kit at the University of North Texas Health Science Center Genomics Core. A total of 20 samples were sequenced.

### Processing of smallRNA-seq data

We used FastQC to assess the quality of small RNA-seq data [33]. Adapter sequences were removed using *Trimmomatic* [34]. On average, 3% of the raw reads were filtered out due to low quality score. Furthermore, we only kept reads of 16–30 nt in length for further analysis to focus on reads that come from miRNAs. We used *bowtie2* [35] with—*very-sensitive-local* parameter to map the reads to the *Arabidopsis thaliana* mature miRNA reference transcriptome obtained from mirBase [31]. To compute read counts for mature miRNAs, Samtools [36] was used to process the alignment file and the miRNA annotation file obtained from miRBase. One replicate each for 3-dpi and 4-dpi mock-inoculated samples were eliminated from the analysis due to low read counts.

### Computing differentially expressed miRNAs

We computed differentially expressed (DE) miRNAs between infected (3-dpi and 4-dpi combined) and mock-inoculated (3-dpi and 4-dpi combined). To observe differential expressions at each time point, we also computed DE miRNAs between 3-dpi infected vs. mock-inoculated and 4-dpi infected vs. mock-inoculated. Because the expression profile did not change between 3-dpi and 4-dpi for the mock samples, we combined them for the analyses. For all cases, we used DESeq2 [37] with default parameters and reported miRNAs with adjusted p-value ≤ 0.05 as DE. To ensure low expressed miRNAs are not considered for DE analysis, only miRNAs with sum of read counts > 10 across all samples were used.

### Functional analysis of the DE miRNAs target genes

To investigate the functions of identified DE miRNAs, we performed Gene Ontology (GO) enrichment analysis for the known target genes of DE miRNAs obtained from 3-dpi infected and mock-inoculated and 4-dpi infected vs. mock-inoculated separately. MiRNA target genes were obtained from the publicly available databases TarDB [38], mirTarBase v9 [39] and TarBase v8 [40]. We utilized AmiGO [41] with default parameters and FDR cutoff < 0.05 to obtain significantly enriched GO terms associated with DE miRNA’s target genes.

Quantitative RT-PCR (qRT-PCR) analysis of four miRNA target genes was conducted to confirm the inverse correlation between miRNA levels and expression of their target genes. One microgram of the same total RNA extracted from plants infected with *F*. *graminearum* or mock-inoculated was treated with DNAse and cDNA was synthesized using LunaSCript RT MasterMix kit (New England Biolabs), with random hexamer and oligo-dT primers. Quantitative PCR was carried out in 96-well plates using gene-specific primers and a VWR qPCR Master Mix (Avantor) for 40 cycles on a QuantStudio 6 Flex Real-Time PCR System (ThermoFisher). Relative expression was calculated as described in Hellemans *et al*. [42] using the *EF-1α* gene (*AT5G60390*) as endogenous reference.

## Results and discussion

### Determination of the timing for sampling

As the goal of this work was to detect early changes in miRNA expression in response to *F*. *graminearum* infection, we first carried out an experiment to determine the time from inoculation to the onset of symptoms. Two 5-week-old Arabidopsis plants were infiltrated with *F*. *graminearum* and plants were visually inspected twice a day to detect early signs of symptoms. At 5 days, visible chlorosis becomes evident on the inoculated leaves (S1 Fig); therefore, we chose 3- and 4-days post inoculation (dpi) as the timepoints to detect early changes in the miRNA transcriptome.

### Differentially expressed miRNAs

**Fig 1:**
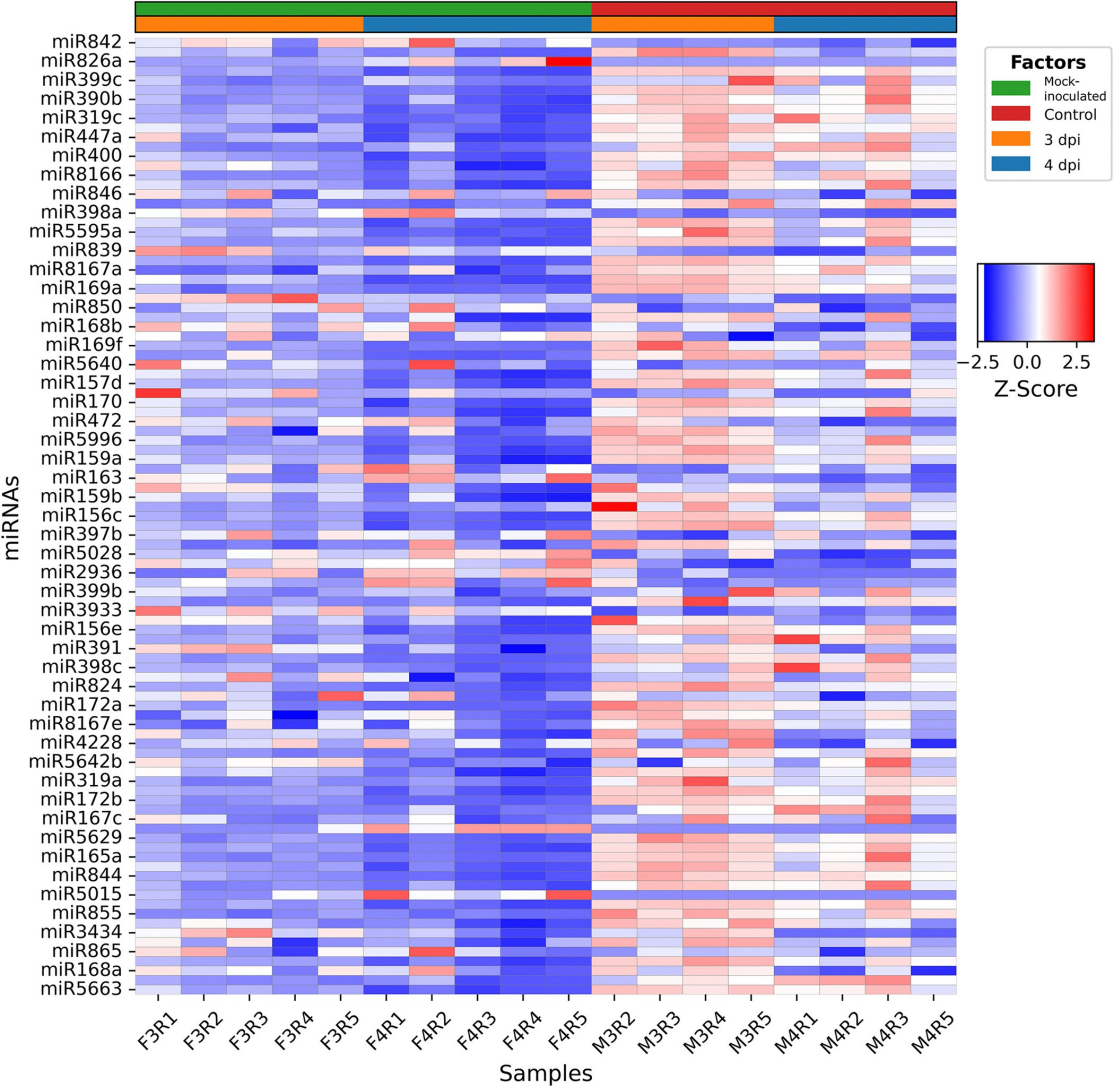
Heatmap of DE miRNAs between infected (3-dpi and 4-dpi combined) and mock-inoculated (3-dpi and 4-dpi combined). Y-axis represents DE miRNAs, and x-axis represents infected and mock-inoculated samples.

RNA-seq analysis of miRNAs was conducted on leaf samples collected from Arabidopsis plants infected with the fungal pathogen *F*. *graminearum* and mock-inoculated at 3-dpi and 4-dpi. We initially conducted an analysis to determine miRNAs that were differentially expressed (DE) when comparing all samples infected with *F*. *graminearum* with those mock-inoculated. Using this approach, a total of 93 miRNAs (out of 428 precursor miRNAs) were identified to be DE between samples infected with *F*. *graminearum* and mock-inoculated samples considering 3-dpi and 4-dpi samples together. The complete list of DE miRNAs identified in 3-dpi and 4-dpi vs. mock is provided in S1 Table. Out of these 93 DE miRNAs, 53 miRNAs were up-regulated, and 40 miRNAs were down-regulated (Fig 1).

**Fig 2:**
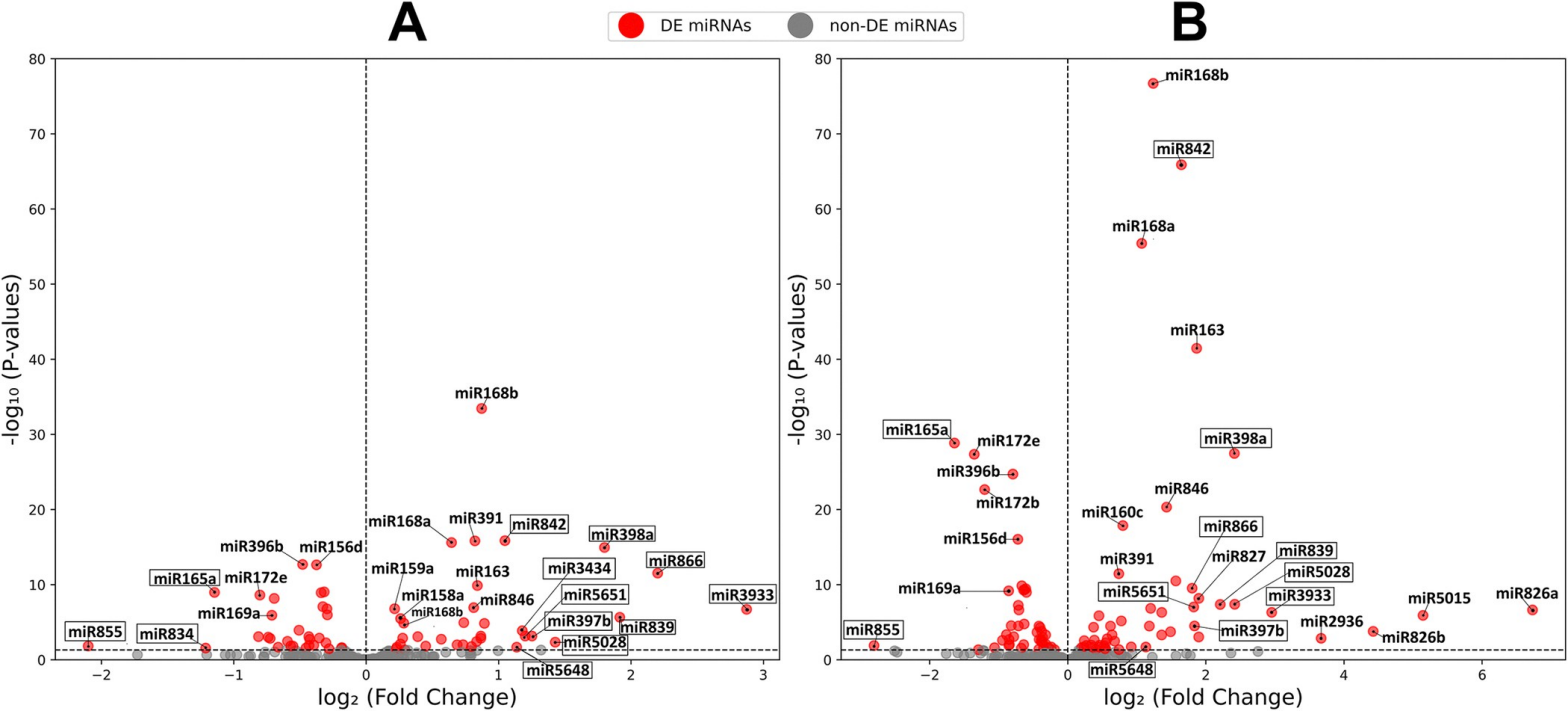
Volcano plot for the DE miRNAs identified in (A) 3-dpi infected vs. mock-inoculated, and (B) 4-dpi infected vs. mock-inoculated. MiRNAs in rectangular boxes represent common DE miRNAs between 3-dpi infected vs. mock-inoculated and 4-dpi infected vs. mock-inoculated with ≥2-fold change.

**Fig 3:**
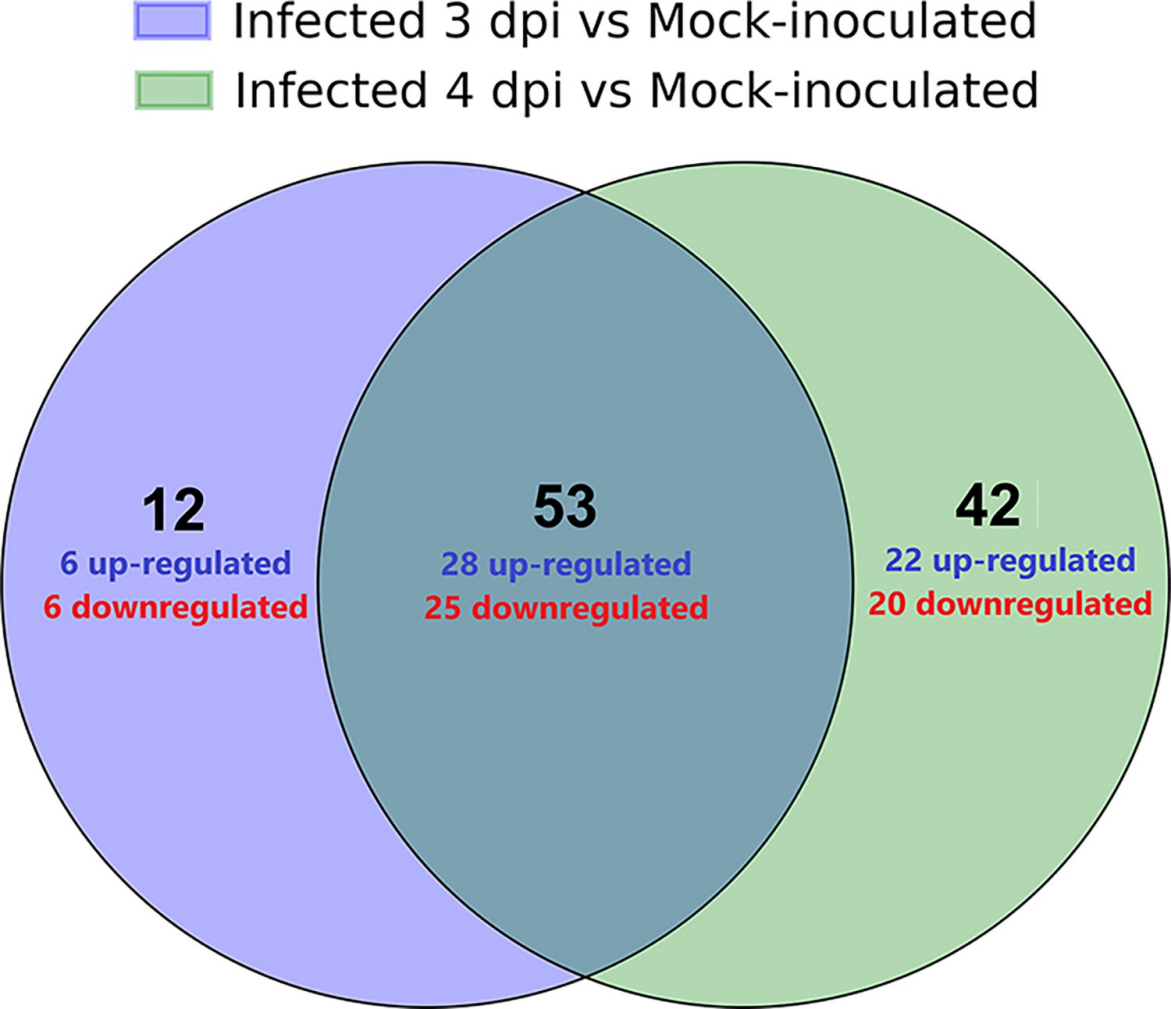
Venn diagram of DE miRNAs identified in 3-dpi infected vs. Mock-inoculated and 4-dpi vs. Mock-inoculated comparisons.

To assess miRNA expression changes at 3-dpi and 4-dpi vs. mock-inoculated individually, we also conducted DE analysis for each time point separately. As we did not observe significant differences in the expression of miRNAs in mock-inoculated samples between 3-dpi and 4-dpi, we combined all the mock-inoculated samples into a single set in this analysis (Fig 2). The complete list of DE miRNAs identified in both 3-dpi infected vs. mock-inoculated and 4-dpi infected vs. mock-inoculated are provided in S2 and S3 Tables, respectively. In total, there were 65 DE miRNAs when 3-dpi infected samples were compared to the mock-inoculated, whereas 95 miRNAs were DE between 4-dpi infected samples and the mock-inoculated. There were 53 DE miRNAs common to both sampled time points (Fig 3). Among the 3-dpi fungal infected samples, ten miRNAs (miR842, miR5648, miR3434, miR5651, miR397b, miR5028, miR398a, miR839, miR866, and miR3933) showed at least 2-fold up-regulation relative to the mock-inoculated samples, whereas only three miRNAs (miR165a, miR855, miR834) showed ≥2-fold down-regulation (S2 Table). In 4-dpi fungal infected samples, there were 31 DE miRNAs with ≥2-fold up- or down-regulation (6 down-regulated and 25 up-regulated), which included 11 out of the 13 DE miRNAs identified in 3-dpi infected samples (S3 Table).

Of the ten miRNAs that were ≥2-fold up-regulated in 3-dpi infected samples, miR398a was up-regulated almost 3.5-fold and this miRNA has been shown to respond to biotic and abiotic stress as part of a general stress response mechanism by plants [43]. MiR397a has been implicated in controlling various plant processes by targeting the mRNAs of genes such as laccases (lignin biosynthesis), β-tubulin, CKB3 (circadian rhythm), and other genes involved in plant growth and development [44, 45]; it is expected that miR397b (2.4-fold up-regulation in this study) also targets these protein-coding genes, as the mature miRNA sequences for miR397a and miR397b only differ by a single nucleotide. MiR397a expression has also been shown to respond to copper and cadmium toxicity [46]. Most of the remaining >2-fold up-regulated miRNAs at 3-dpi, miR842, miR5648, miR3434, miR5651, miR5028, miR3933, and miR839 have also been identified in other transcriptome analyses, however they appeared relatively recently in evolution and their functions have not yet been completely elucidated. Both miR3933 (formerly called miR2328) and miR839 seem to associate with AGO4 (instead of the more common AGO1-associated miRNAs) and can generate small interfering RNAs (siRNAs) that influence DNA methylation of their target genes [47, 48]. Therefore, it is possible that these miRNAs participate in the plant stress response via a different mechanism than the typical RISC-mediated down-regulation of target genes.

MiR855 was down-regulated approximately 4.3-fold in 3-dpi and 7-fold in 4-dpi infected samples compared to mock-inoculated samples. A miR855 homolog in wheat (Ta-miR855) was identified using a computational approach and predicted to target a MYB transcriptional activator and a transporter in this species in response to cold and salt stresses [49]. Therefore, it is possible that genetic manipulation of miR855 expression, either by traditional breeding or biotechnological approaches, may have direct application in developing wheat cultivars with improved resistance to *F*. *graminearum*, as well as other stresses.

MiR826a and miR5015 showed the highest up-regulation in 4-dpi infected plants of 106-fold and 35-fold, respectively (Fig 2 and S3 Table). MiR826 is a recently evolved miRNA, which seems to only be present in *A*. *thaliana*. This miRNA was shown to be up-regulated in Arabidopsis plants grown under nutrient deficiency conditions, specifically carbon, nitrogen and sulfur [50, 51], whereas it is down-regulated early during exposure of plant roots to toxic levels of copper and cadmium [46]. Its up-regulation under nitrogen starvation conditions has been linked with a decrease in the synthesis of methionine-derived glucosinolates; these specialized metabolites, which are produced by cruciferous plants, are known to be involved in defense against biotic stressors [51]. Therefore, our results showing that miR826a is highly up-regulated in response to fungal infection is surprising and warrants future investigation. Nevertheless, identifying the downstream protein-coding gene(s) targeted by miR826a could provide an avenue for plant genetic modifications aimed at improving their resilience to stress.

MiR5015 was previously identified by a computational approach in mint (*Mentha* spp.) plants to be involved in the development of trichomes associated with the synthesis of essential oils [52], which are known to provide plant protection against herbivores and fungal pathogens [53]. There is also some evidence for a role of miR5015 in suppressing phenylalanine ammonia-lyase (PAL), the enzyme that catalyzes the first step in the phenylpropanoid pathway, which is responsible for synthesis of several defense compounds [54]. Finally, miR5015 was recently shown in Arabidopsis pollen to target the transcription factor EIN3, involved in plant responses to the stress hormone ethylene [55]. It will be interesting to test the expression of putative targets of miR5015 to identify its role during *F*. *graminearum* infection of Arabidopsis plants.

### Functional analysis of the DE miRNA’s target genes

**Fig 4:**
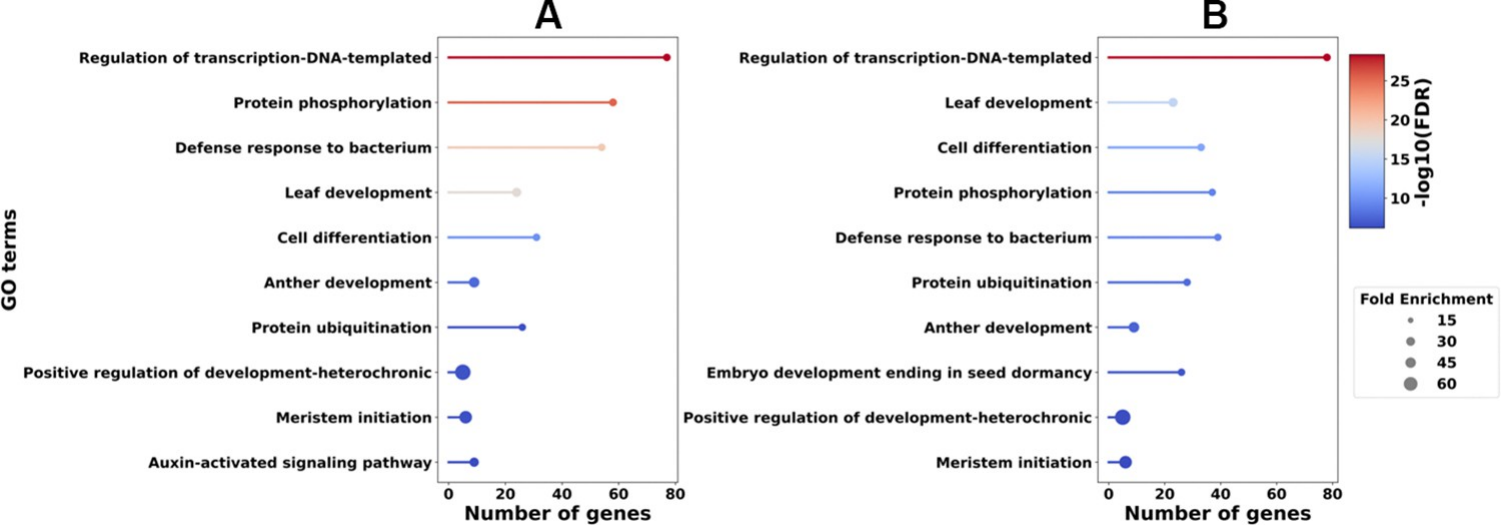
Top 10 enriched GO Biological Process terms for (A) the target genes of DE miRNAs obtained from 3-dpi infected vs. mock-inoculated (B) the target genes of DE miRNAs obtained from 4-dpi infected vs. mock-inoculated.

We performed a Gene Ontology (GO) enrichment analysis to gain an insight over the functional roles of genes that are targeted by the DE miRNAs. We obtained 774 and 724 target genes of DE miRNAs in 3-dpi infected vs. mock-inoculated and 4-dpi infected vs. mock-inoculated from public databases, respectively. We performed GO enrichment analysis of these target genes separately and obtained a total of 189 GO Biological Process (BP), 46 GO Cellular Component (CC), and 80 GO Molecular Function (MF) terms (Table 1), which correspond to the union of GO terms that were enriched from 3-dpi infected vs. mock-inoculated and 4-dpi infected vs. mock-inoculated. Over 85% of these GO terms appeared in both enrichment analyses. Enriched GO BP terms included stress-related terms such as defense response to bacterium, regulation of DNA-templated transcription, defense response, leaf senescence, response to abscisic acid, and positive regulation of programmed cell death. Fig 4 illustrates the top ten BP enriched GO terms in 3-dpi infected vs. mock-inoculated (Fig 4A) and 4-dpi infected vs. mock-inoculated (Fig 4B) comparisons with a False Discovery Rate (FDR) of 0.05 or less. S4–S6 Tables contain the full list of enriched BP, CC and MF GO terms, respectively.

**Table 1:** Number of enriched GO terms for the target genes of DE miRNAs. The first two columns show the unique number of enriched GO terms for each experiment, followed by the number of common GO terms. *dpi*: days post inoculation.

	3-dpi *vs*. mock-inoculated	4-dpi *vs*. mock-inoculated	Common
**Biological Process**	22	17	167
**Molecular Function**	4	2	69
**Cellular Component**	6	5	40

Unsurprisingly, plants infected with *F*. *graminearum* appear to change expression of genes related to transcriptional control and to reprogramming development, as well as synthesis of proteins involved in mitigating the effects of oxidative stress. The observed enrichment for 1,3-β-D-glucan synthase complex likely reflects synthesis of callose (a polymer containing β-1,3-linked glucose) by plants to restrict penetration by the fungus; these polymers are also typical of fungal cell walls [56]. Callose synthesis and deposition in various tissues has been associated with responses to both biotic and abiotic stresses in plants [57, 58].

### Expression changes of miRNA’s target genes

**Fig 5:**
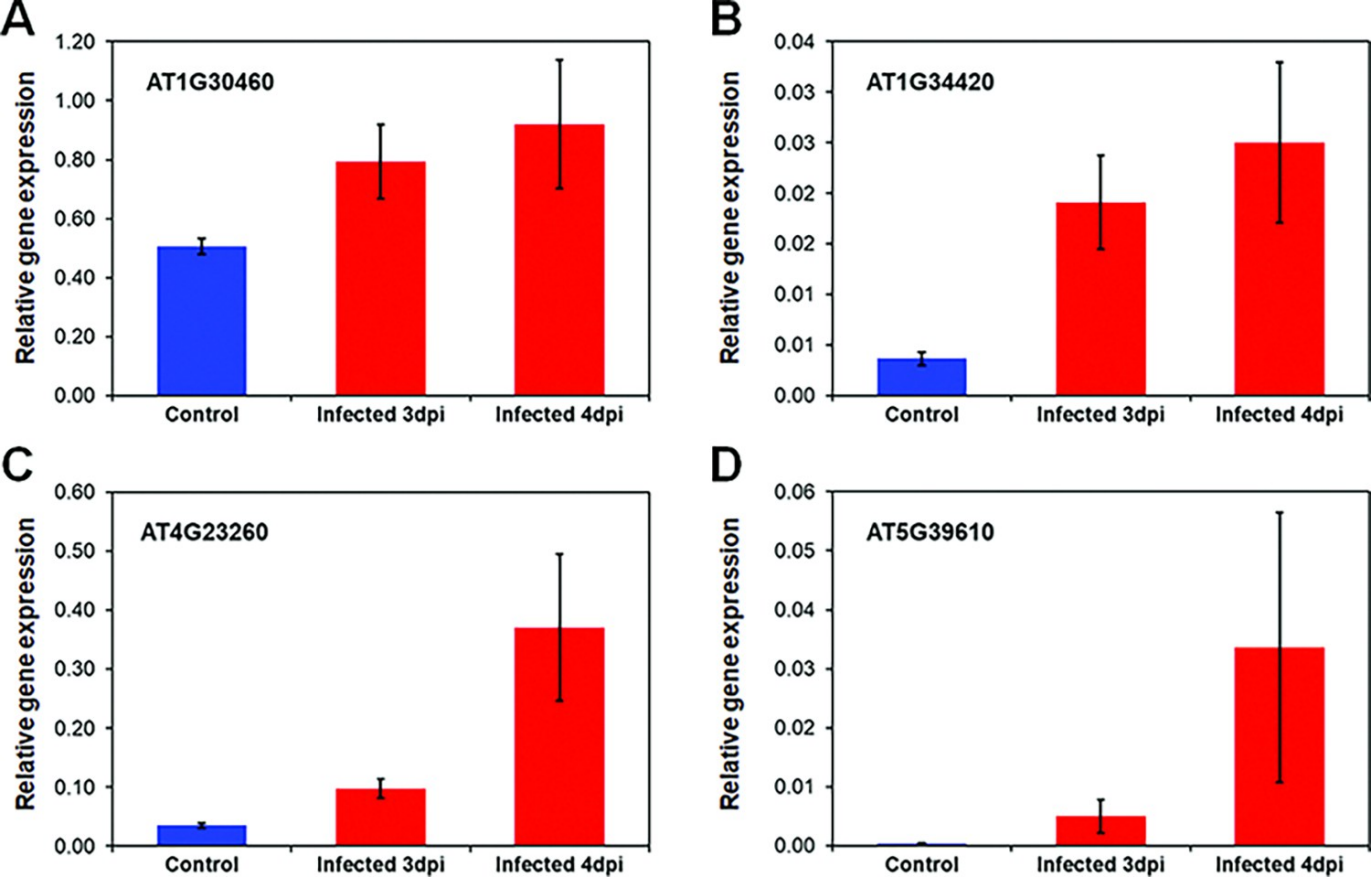
Expression of target genes of top DE miRNAs in 3-dpi and 4-dpi fungal infected, and mock-inoculated plants. (A) *AT1G30460*; (B) *AT1G34420*, *BTL2*; (C) *AT4G23260*, *Cysteine-rich receptor-like protein kinase 18*; (D) *AT5G39610*, *ANAC092*. The expression of all genes was normalized to that of EF1α. The data are shown as mean ± SEM (standard error, n = 4).

To confirm that the changes in miRNA expression observed in response to *F*. *graminearum* infection result in the corresponding reciprocal modulation of expression of their target genes, we conducted RT-qPCR analyses of a select number of target genes from significantly enriched GO terms (Fig 5). Gene *AT1G30460* encodes AtCPSF30, a subunit of a cleavage and polyadenylation specificity factor, and is a positive regulator of programmed cell death (PCD) [59]. This gene is predicted to be targeted by miR157c, which showed 1.4-fold down-regulation in 4-dpi infected samples relative to the mock-inoculated samples. AtCPSF30 showed similar corresponding up-regulation of 1.6-fold at 3-dpi and 1.8-fold at 4-dpi (Fig 5A).

Gene *AT1G34420* encodes a receptor kinase protein called Bak-to-Life 2 (BTL2), which triggers autoimmunity through activation of the Ca^2+^ channel CNGC20 in a kinase-dependent manner when the PRR (pattern recognition receptor) co-receptors BAK1/SER4 are perturbed [60]. *BTL2* was up-regulated almost 7-fold in 4-dpi infected samples (Fig 5B), and is predicted to be targeted by miR396b, which was down-regulated 1.6-fold in 4-dpi samples relative to the mock-inoculated samples.

Gene *AT4G23260* encodes a cysteine-rich receptor-like protein kinase (AtCRK18) and is a predicted target of miR172c (down-regulated 3.5-fold). CRKs have been shown to play a role in disease resistance and plant cell death [61]. CRK18 was up-regulated more than 10-fold in 4-dpi infected plants relative to the mock-inoculated plants (Fig 5C).

Gene *AT5G39610* encodes a NAC-domain transcription factor (ANAC092), which positively regulates aging-induced cell death and senescence in leaves. This gene has been shown to be upregulated in response to salt stress in Arabidopsis, as well as in response to ABA, ACC and NAA treatment [62]. ANAC092 is targeted by miR164b, which was down-regulated approximately 1.5-fold in 4-dpi samples. This modest down-regulation of miR164b resulted in a large 91-fold up-regulation of its target gene *ANAC092* (Fig 5D).

The changes observed in all four miRNA target genes tested were consistent with an up-regulation of the target protein-coding genes when their corresponding predicted miRNAs are down-regulated in response to fungal infection and reflect a plant response to limit penetration of the pathogen. Programmed cell death (PCD) is a genetically controlled process that occurs during both plant development and defense responses [63, 64]. Hypersensitive response is a form of PCD in plants that is linked to resistance to several pathogens, including fungi such as *F*. *graminearum*. Up-regulation of AtCPSF30, AtCRK18 and ANAC092 is thus consistent with plants inducing PCD to arrest pathogen growth [65]. BTL2 is a recently identified LRR-RK (leucine-rich repeat receptor kinase) protein that functions as a type of surveillance system that activates multiple signaling pathways to modulate immunity in plants. Increased BTL2 expression has been shown to trigger autoimmunity, which is kept in check by BAK1 phosphorylation of BTL2 [60].

## Conclusions

In this study, we have identified several miRNAs whose expression vary significantly during the early (*i*.*e*., pre-symptomatic) stages of infection of *Arabidopsis thaliana* plants by the fungal pathogen *Fusarium graminearum*. To the best of our knowledge, this is the first study to investigate early-stage miRNA expression changes in Arabidopsis plants infected by the fungal pathogen *F*. *graminearum*. Overall, our results indicate that during the early stages of this infection, Arabidopsis plants respond by changing the levels of miRNAs that control expression of proteins involved in restricting the pathogen’s penetration into the plant tissue, especially by triggering a hypersensitive response. These results also suggest that miRNAs may indeed constitute a first line of defense deployed by plants to cope with stresses. Future studies to uncover the mechanistic roles of the DE miRNAs identified here, *e*.*g*., miRNA855, may inform new approaches to develop plant cultivars with improved resistance to *F*. *graminearum* infection.

## Supporting information

S1 Fig Preliminary inoculation of Arabidopsis plants with *Fusarium graminearum* to determine the time for development of symptoms. (PDF)

S1 Table List of differentially expressed miRNAs in 3-dpi and 4-dpi fungal- vs. Mock-inoculated plants. (XLSX)

S2 Table List of differentially expressed miRNAs in 3-dpi fungal- vs. Mock-inoculated plants. (XLSX)

S3 Table List of differentially expressed miRNAs in 4-dpi fungal- vs. Mock-inoculated plants. (XLSX)

S4 Table Significantly enriched Biological Process (BP) Gene Ontology (GO) terms for the target genes of DE miRNAs in 3-dpi fungal- vs. Mock-inoculated plants, or target genes of DE miRNAs in 4-dpi fungal- vs. Mock-inoculated plants (False Discovery Rate (FDR) ≤ 0.05). NS: Not significant.
(XLSX)

S5 Table Significantly enriched Molecular Function (MF) Gene Ontology (GO) terms for the target genes of DE miRNAs in 3-dpi fungal- vs. Mock-inoculated plants, or target genes of DE miRNAs in 4-dpi fungal- vs. Mock-inoculated plants (False Discovery Rate (FDR) ≤ 0.05). NS: Not significant.
(XLSX)

S6 Table Significantly enriched Cellular Component (CC) Gene Ontology (GO) terms for the target genes of DE miRNAs in 3-dpi fungal- vs. Mock-inoculated plants, or target genes of DE miRNAs in 4-dpi fungal- vs. Mock-inoculated plants (False Discovery Rate (FDR) ≤ 0.05). NS: Not significant.
(XLSX)

## References

[1] Khraiwesh B, Zhu J, Zhu J. Role of miRNAs and siRNAs in biotic and abiotic stress responses of plants. Biochimica et Biophysica Acta (BBA)—Gene Regulatory Mechanisms. 2012;1819: 137–148. doi: 10.1016/j.bbagrm.2011.05.001 21605713 PMC3175014

[2] Shukla LI, Chinnusamy V, Sunkar R. The role of microRNAs and other endogenous small RNAs in plant stress responses. Biochimica et Biophysica Acta (BBA)—Gene Regulatory Mechanisms. 2008;1779: 743–748. doi: 10.1016/j.bbagrm.2008.04.004 18457682

[3] Islam W, Qasim M, Noman A, Adnan M, Tayyab M, Farooq TH, et al. Plant microRNAs: Front line players against invading pathogens. Microbial pathogenesis. 2018;118: 9–17. doi: 10.1016/j.micpath.2018.03.008 29524548

[4] Borges F, Martienssen RA. The expanding world of small RNAs in plants. Nat Rev Mol Cell Biol. 2015;16: 727–741. doi: 10.1038/nrm4085 26530390 PMC4948178

[5] Weiberg A, Jin H. Small RNAs—the secret agents in the plant–pathogen interactions. Current Opinion in Plant Biology. 2015;26: 87–94. doi: 10.1016/j.pbi.2015.05.033 26123395 PMC4573252

[6] Huang J, Yang M, Zhang X. The function of small RNAs in plant biotic stress response. J Integr Plant Biol. 2016;58: 312–327. doi: 10.1111/jipb.12463 26748943

[7] Huang J, Yang M, Lu L, Zhang X. Diverse Functions of Small RNAs in Different Plant-Pathogen Communications. Front Microbiol. 2016;7: 1552. doi: 10.3389/fmicb.2016.01552 27757103 PMC5048074

[8] Chapman EJ, Prokhnevsky AI, Gopinath K, Dolja VV, Carrington JC. Viral RNA silencing suppressors inhibit the microRNA pathway at an intermediate step. Genes Dev. 2004;18: 1179–1186. doi: 10.1101/gad.1201204 15131083 PMC415642

[9] Chen J, Li WX, Xie D, Peng JR, Ding SW. Viral virulence protein suppresses RNA silencing-mediated defense but upregulates the role of microrna in host gene expression. Plant Cell. 2004;16: 1302–1313. doi: 10.1105/tpc.018986 15100397 PMC423217

[10] Navarro L, Jay F, Nomura K, He SY, Voinnet O. Suppression of the microRNA pathway by bacterial effector proteins. Science. 2008;321: 964–967. doi: 10.1126/science.1159505 18703740 PMC2570098

[11] Uppalapati SR, Marek SM, Lee HK, Nakashima J, Tang Y, Sledge MK, et al. Global gene expression profiling during Medicago truncatula-Phymatotrichopsis omnivora interaction reveals a role for jasmonic acid, ethylene, and the flavonoid pathway in disease development. Mol Plant Microbe Interact. 2009;22: 7–17. doi: 10.1094/MPMI-22-1-0007 19061398

[12] Salvador-Guirao R, Baldrich P, Weigel D, Rubio-Somoza I, San Segundo B. The MicroRNA miR773 Is Involved in the Arabidopsis Immune Response to Fungal Pathogens. Molecular plant-microbe interactions. 2018;31: 249–259. doi: 10.1094/MPMI-05-17-0108-R 28990488

[13] Gao X, Cui Q, Cao QZ, Liu Q, He HB, Zhang DM, et al. Transcriptome-Wide Analysis of Botrytis elliptica Responsive microRNAs and Their Targets in Lilium Regale Wilson by High-Throughput Sequencing and Degradome Analysis. Front Plant Sci. 2017;8: 753. doi: 10.3389/fpls.2017.00753 28572808 PMC5435993

[14] Thiebaut F, Grativol C, Hemerly AS, Ferreira PCG. MicroRNA Networks in Plant-Microorganism Interactions. Tropical Plant Biol. 2015;8: 40–50. doi: 10.1007/s12042-015-9149-9

[15] Fahlgren N, Howell MD, Kasschau KD, Chapman EJ, Sullivan CM, Cumbie JS, et al. High-throughput sequencing of Arabidopsis microRNAs: evidence for frequent birth and death of MIRNA genes. PLoS One. 2007;2: e219. doi: 10.1371/journal.pone.0000219 17299599 PMC1790633

[16] Jagadeeswaran G, Zheng Y, Li YF, Shukla LI, Matts J, Hoyt P, et al. Cloning and characterization of small RNAs from Medicago truncatula reveals four novel legume-specific microRNA families. New Phytol. 2009;184: 85–98. doi: 10.1111/j.1469-8137.2009.02915.x 19555436

[17] Barah P, Winge P, Kusnierczyk A, Tran DH, Bones AM. Molecular signatures in Arabidopsis thaliana in response to insect attack and bacterial infection. PLoS One. 2013;8: e58987. doi: 10.1371/journal.pone.0058987 23536844 PMC3607608

[18] Zhao H, Sun R, Albrecht U, Padmanabhan C, Wang A, Coffey MD, et al. Small RNA profiling reveals phosphorus deficiency as a contributing factor in symptom expression for citrus huanglongbing disease. Mol Plant. 2013;6: 301–310. doi: 10.1093/mp/sst002 23292880 PMC3716302

[19] Zhang W, Gao S, Zhou X, Chellappan P, Chen Z, Zhou X, et al. Bacteria-responsive microRNAs regulate plant innate immunity by modulating plant hormone networks. Plant Mol Biol. 2011;75: 93–105. doi: 10.1007/s11103-010-9710-8 21153682 PMC3005105

[20] Nobori T, Velásquez AC, Wu J, Kvitko BH, Kremer JM, Wang Y, et al. Transcriptome landscape of a bacterial pathogen under plant immunity. Proc Natl Acad Sci USA. 2018;115: E3055–E3064. doi: 10.1073/pnas.1800529115 29531038 PMC5879711

[21] Irani S, Trost B, Waldner M, Nayidu N, Tu J, Kusalik AJ, et al. Transcriptome analysis of response to Plasmodiophora brassicae infection in the Arabidopsis shoot and root. BMC Genomics. 2018;19: 23. doi: 10.1186/s12864-017-4426-7 29304736 PMC5756429

[22] Zhu Q, Stephen S, Kazan K, Jin G, Fan L, Taylor J, et al. Characterization of the defense transcriptome responsive to Fusarium oxysporum-infection in Arabidopsis using RNA-seq. Gene. 2013;512: 259–266. doi: 10.1016/j.gene.2012.10.036 23107761

[23] Howard BE, Hu Q, Babaoglu AC, Chandra M, Borghi M, Tan X, et al. High-Throughput RNA Sequencing of Pseudomonas-Infected Arabidopsis Reveals Hidden Transcriptome Complexity and Novel Splice Variants. PLoS ONE. 2013;8: e74183–e74183. doi: 10.1371/journal.pone.0074183 24098335 PMC3788074

[24] De Vos M, Van Oosten VR, Van Poecke RM, Van Pelt JA, Pozo MJ, Mueller MJ, et al. Signal signature and transcriptome changes of Arabidopsis during pathogen and insect attack. Mol Plant Microbe Interact. 2005;18: 923–937. doi: 10.1094/MPMI-18-0923 16167763

[25] Kurubanjerdjit N, Tsai JJ, Huang CH, Ng KL. Disturbance of Arabidopsis thaliana microRNA-regulated pathways by Xcc bacterial effector proteins. Amino Acids. 2014;46: 953–961. doi: 10.1007/s00726-013-1646-2 24385242

[26] Djami-Tchatchou A, Dubery IA. miR393 regulation of lectin receptor-like kinases associated with LPS perception in Arabidopsis thaliana. Biochem Biophys Res Commun. 2019;513: 88–92. doi: 10.1016/j.bbrc.2019.03.170 30940349

[27] Dweba CC, Figlan S, Shimelis HA, Motaung TE, Sydenham S, Mwadzingeni L, et al. Fusarium head blight of wheat: Pathogenesis and control strategies. Crop protection. 2017;91: 114–122. doi: 10.1016/j.cropro.2016.10.002

[28] Wegulo SN, Baenziger PS, Hernandez Nopsa J, Bockus WW, Hallen-Adams H. Management of Fusarium head blight of wheat and barley. Crop protection. 2015;73: 100–107. doi: 10.1016/j.cropro.2015.02.025

[29] Sobrova P, Adam V, Vasatkova A, Beklova M, Zeman L, Kizek R. Deoxynivalenol and its toxicity. Interdisciplinary toxicology. 2010;3: 94–99. doi: 10.2478/v10102-010-0019-x 21217881 PMC2984136

[30] Kazan K, Gardiner DM. Transcriptomics of cereal–Fusarium graminearum interactions: what we have learned so far. Molecular plant pathology. 2018;19: 764–778. doi: 10.1111/mpp.12561 28411402 PMC6638174

[31] Griffiths-Jones S, Grocock RJ, van Dongen S, Bateman A, Enright AJ. miRBase: microRNA sequences, targets and gene nomenclature. Nucleic acids research. 2006;34: D140–D144. doi: 10.1093/nar/gkj112 16381832 PMC1347474

[32] Nalam V, Sarowar S, Shah J. Establishment of a Fusarium graminearum Infection Model in Arabidopsis thaliana Leaves and Floral Tissues. BIO-PROTOCOL. 2016;6. doi: 10.21769/bioprotoc.1877

[33] Andrews S. A quality control tool for high throughput sequence data. 2010. Available: https://www.bioinformatics.babraham.ac.uk/projects/fastqc/.

[34] Bolger AM, Lohse M, Usadel B. Trimmomatic: a flexible trimmer for Illumina sequence data. Bioinformatics. 2014;30: 2114–2120. doi: 10.1093/bioinformatics/btu170 24695404 PMC4103590

[35] Langmead B, Salzberg SL. Fast gapped-read alignment with Bowtie 2. Nat Methods. 2012;9: 357–359. doi: 10.1038/nmeth.1923 22388286 PMC3322381

[36] Danecek P, Bonfield JK, Liddle J, Marshall J, Ohan V, Pollard MO, et al. Twelve years of SAMtools and BCFtools. GigaScience. 2021;10: giab008. doi: 10.1093/gigascience/giab008 33590861 PMC7931819

[37] Love MI, Huber W, Anders S. Moderated estimation of fold change and dispersion for RNA-seq data with DESeq2. Genome Biol. 2014;15: 550. doi: 10.1186/s13059-014-0550-8 25516281 PMC4302049

[38] Liu J, Liu X, Zhang S, Liang S, Luan W, Ma X. TarDB: an online database for plant miRNA targets and miRNA-triggered phased siRNAs. BMC genomics. 2021;22: 348. doi: 10.1186/s12864-021-07680-5 33985427 PMC8120726

[39] Huang H, Lin Y, Cui S, Huang Y, Tang Y, Xu J, et al. miRTarBase update 2022: an informative resource for experimentally validated miRNA–target interactions. Nucleic Acids Research. 2022;50: 222. doi: 10.1093/nar/gkab1079 34850920 PMC8728135

[40] Karagkouni D, Paraskevopoulou MD, Chatzopoulos S, Vlachos IS, Tastsoglou S, Kanellos I, et al. DIANA-TarBase v8: a decade-long collection of experimentally supported miRNA-gene interactions. Nucleic acids research. 2018;46: D239–D245. doi: 10.1093/nar/gkx1141 29156006 PMC5753203

[41] Carbon S, Ireland A, Mungall CJ, Shu S, Marshall B, Lewis S. AmiGO: online access to ontology and annotation data. Bioinformatics. 2009; 25: 288–289. doi: 10.1093/bioinformatics/btn615 19033274 PMC2639003

[42] Hellemans J, Mortier G, De Paepe A, Speleman F, Vandesompele J. qBase relative quantification framework and software for management and automated analysis of real-time quantitative PCR data. Genome Biology. 2007;8: R19–R19. doi: 10.1186/gb-2007-8-2-r19 17291332 PMC1852402

[43] Zhu C, Ding Y, Liu H. MiR398 and plant stress responses. Physiologia Plantarum. 2011;143: 1–9. doi: 10.1111/j.1399-3054.2011.01477.x 21496029

[44] Huang S, Zhou J, Gao L, Tang Y. Plant miR397 and its functions. Functional plant biology: FPB. 2021;48: 361–370. doi: 10.1071/FP20342 33333000

[45] Feng Y, Yu Y, Zhou Y, Yang Y, Lei M, Lian J, et al. A Natural Variant of miR397 Mediates a Feedback Loop in Circadian Rhythm. Plant physiology (Bethesda). 2020;182: 204–214. doi: 10.1104/pp.19.00710 31694901 PMC6945863

[46] Gielen H, Remans T, Vangronsveld J, Cuypers A. Toxicity responses of Cu and Cd: the involvement of miRNAs and the transcription factor SPL7. BMC plant biology. 2016;16: 145. doi: 10.1186/s12870-016-0830-4 27352843 PMC4924269

[47] Pegler JL, Oultram JMJ, Mann CWG, Carroll BJ, Grof CPL, Eamens AL. Miniature Inverted-Repeat Transposable Elements: Small DNA Transposons That Have Contributed to Plant MICRORNA Gene Evolution. Plants (Basel). 2023;12: 1101. doi: 10.3390/plants12051101 36903960 PMC10004981

[48] Chellappan P, Xia J, Zhou X, Gao S, Zhang X, Coutino G, et al. siRNAs from miRNA sites mediate DNA methylation of target genes. 2010. doi: 10.1093/nar/gkq590 20621980 PMC2978365

[49] Pandey B, Gupta OP, Pandey DM, Sharma I, Sharma P. Identification of new stress-induced microRNA and their targets in wheat using computational approach. Plant signaling & behavior. 2013;8: e23932. doi: 10.4161/psb.23932 23511197 PMC3906146

[50] Liang G, He H, Yu D. Identification of Nitrogen Starvation-Responsive MicroRNAs in Arabidopsis thaliana. PLoS ONE. 2012;7: e48951–e48951. doi: 10.1371/journal.pone.0048951 23155433 PMC3498362

[51] He H, Liang G, Li Y, Wang F, Yu D. Two Young MicroRNAs Originating from Target Duplication Mediate Nitrogen Starvation Adaptation via Regulation of Glucosinolate Synthesis in Arabidopsis thaliana. Plant Physiology. 2014;164: 853–865. doi: 10.1104/pp.113.228635 24367020 PMC3912111

[52] Singh N, Srivastava S, Shasany AK, Sharma A. Identification of miRNAs and their targets involved in the secondary metabolic pathways of Mentha spp. Computational biology and chemistry. 2016;64: 154–162. doi: 10.1016/j.compbiolchem.2016.06.004 27376499

[53] Nazzaro F, Fratianni F, Coppola R, Feo VD. Essential Oils and Antifungal Activity. Pharmaceuticals. 2017;10: 86. doi: 10.3390/ph10040086 29099084 PMC5748643

[54] Singh N, Srivastava S, Sharma A. Identification and analysis of miRNAs and their targets in ginger using bioinformatics approach. Gene. 2016;575: 570–576. doi: 10.1016/j.gene.2015.09.036 26392033

[55] Oliver C, Annacondia ML, Wang Z, Jullien PE, Slotkin RK, Köhler C, et al. The miRNome function transitions from regulating developmental genes to transposable elements during pollen maturation. The Plant cell. 2022;34: 784–801. doi: 10.1093/plcell/koab280 34755870 PMC8824631

[56] Douglas CM. Fungal β (1, 3)-D-glucan synthesis. Medical Mycology. 2001;39: 55–66. doi: 10.1080/mmy.39.1.55.66 11800269

[57] Luna E, Pastor V, Robert J, Flors V, Mauch-Mani B, Ton J. Callose Deposition: A Multifaceted Plant Defense Response. Molecular Plant-Microbe Interactions. 2011;24: 183–193. doi: 10.1094/MPMI-07-10-0149 20955078

[58] Li N, Lin Z, Yu P, Zeng Y, Du S, Huang L. The multifarious role of callose and callose synthase in plant development and environment interactions. Frontiers in plant science. 2023;14: 1183402. doi: 10.3389/fpls.2023.1183402 37324665 PMC10264662

[59] Bruggeman Q, Garmier M, de Bont L, Soubigou-Taconnat L, Mazubert C, Benhamed M, et al. The Polyadenylation Factor Subunit CLEAVAGE AND POLYADENYLATION SPECIFICITY FACTOR30: A Key Factor of Programmed Cell Death and a Regulator of Immunity in Arabidopsis. Plant physiology (Bethesda). 2014;165: 732–746. doi: 10.1104/pp.114.236083 24706550 PMC4044851

[60] Yu X, Xie Y, Luo D, Liu H, de Oliveira MVV, Qi P, et al. A phospho-switch constrains BTL2-mediated phytocytokine signaling in plant immunity. Cell. 2023;186: 2329–2344.e20. doi: 10.1016/j.cell.2023.04.027 37192618 PMC10281528

[61] Chen K, Du L, Chen Z. Sensitization of defense responses and activation of programmed cell death by a pathogen-induced receptor-like protein kinase in Arabidopsis. Plant Molecular Biology. 2003;53: 61–74. doi: 10.1023/B:PLAN.0000009265.72567.58 14756307

[62] Xi D, Chen X, Wang Y, Zhong R, He J, Shen J, et al. Arabidopsis ANAC092 regulates auxin‐mediated root development by binding to the ARF8 and PIN4 promoters. Journal of integrative plant biology. 2019;61: 1015–1031. doi: 10.1111/jipb.12735 30415491

[63] Valandro F, Menguer PK, Cabreira-Cagliari C, Margis-Pinheiro M, Cagliari A. Programmed cell death (PCD) control in plants: New insights from the Arabidopsis thaliana deathosome. Plant Science. 2020;299: 110603. doi: 10.1016/j.plantsci.2020.110603 32900441

[64] Huysmans M, Lema A S, Coll NS, Nowack MK. Dying two deaths—programmed cell death regulation in development and disease. Current Opinion in Plant Biology. 2017;35: 37–44. doi: 10.1016/j.pbi.2016.11.005 27865098

[65] Birch PRJ, Avrova AO, Dellagi A, Lacomme C, Cruz SS, Lyon GD. Programmed Cell Death in Plants in Response to Pathogen Attack. Chichester, UK: John Wiley & Sons, Ltd; 2018.

