# Supplementary material for: Early changes in microRNA expression in Arabidopsis plants infected with the fungal pathogen Fusarium graminearum
Source: PLoS One. 2025 Feb 6;20(2):e0318532. doi: 10.1371/journal.pone.0318532 (PMC11801585; doi:10.1371/journal.pone.0318532)
Supplement: S1 Fig — (PDF) [file pone.0318532.s001.pdf]

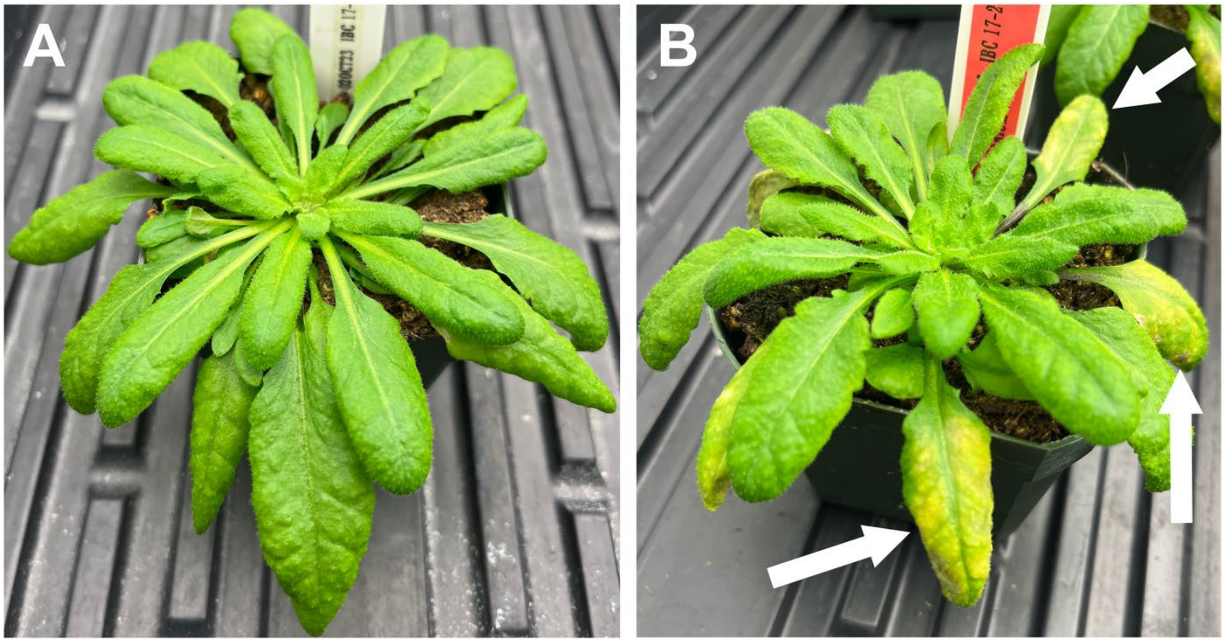

S1 Figure: Preliminary inoculation of Arabidopsis plants with *Fusarium graminearum* to determine the time for development of symptoms. (**A**) Mock inoculated plant at 5 days post inoculation; (**B**) Fungus inoculated plant at 5 days post inoculation. Arrows point to chlorosis symptom on inoculated leaves.
